# Amygdala volumes and associations with socio-emotional competencies in preterm youth: cross-sectional and longitudinal data

**DOI:** 10.1038/s41390-024-03227-y

**Published:** 2024-05-18

**Authors:** Maricé Pereira Camejo, Luciana Escobar Saade, Maria Chiara Liverani, Elda Fischi-Gomez, Laura Gui, Cristina Borradori Tolsa, Russia Ha-Vinh Leuchter, Petra Susan Hüppi, Vanessa Siffredi

**Affiliations:** 1 Division of Development and Growth, Department of Paediatrics, Gynaecology and Obstetrics, Geneva University Hospitals and University of Geneva, Geneva, Switzerland; 2 SensoriMotor, Affective and Social Development Laboratory, Faculty of Psychology and Educational Sciences, University of Geneva, Geneva, Switzerland; 3 Centre for Biomedical Imaging (CIBM), SP CHUV-EPFL Section, Lausanne, Switzerland; 4 Signal processing laboratory 5, Ecole polytechnique fédérale de Lausanne, Geneva, Switzerland; 5 Department of Radiology, University Hospital and University of Lausanne, Lausanne, Switzerland; 6 Neuro-X Institute, Ecole polytechnique fédérale de Lausanne, Geneva, Switzerland; 7 Department of Radiology and Medical Informatics, Faculty of Medicine, University of Geneva, Geneva, Switzerland; 8 Department of Radiology, Lausanne University Hospital and University of Lausanne, Lausanne, Switzerland

## Abstract

**Background:**

Socio-emotional difficulties often result from very preterm (VPT) birth. The amygdala’s developmental trajectory, including its nuclei, has been recognized as a significant factor in observed difficulties. This study aims to assess the relationship between amygdala volume and socio-emotional competencies in VPT children and adolescents.

**Methods:**

Socio-emotional competencies were assessed, and amygdala volumes, including subnuclei, were extracted automatically from structural scans in a cross-sectional cohort of VPT (*n* = 75) and full-term (FT, *n* = 41) aged 6–14 years. Group differences in amygdala volumes were assessed using ANCOVA, and associations with socio-emotional competencies were studied using partial least squares correlation (PLSC). In a VPT subgroup, additional longitudinal data with amygdala volumes at term-equivalent age (TEA) were manually extracted, growth rates calculated, and associations with school-age socio-emotional competencies investigated using PLSC.

**Results:**

Using cross-sectional data at school-age, amygdala volumes displayed comparable developmental patterns between the VPT and the FT groups. Greater volumes were associated with more emotional regulation difficulties in VPT and lower affect recognition competencies in FT. In the longitudinal VPT subgroup, no significant associations were found between amygdala volume trajectory and socio-emotional competencies.

**Conclusion:**

Although our findings suggest typical amygdala development after VPT birth, further research is necessary to elucidate the developmental trajectory of amygdala and the role of resilience factors.

**Impact:**

- In our cohort, amygdala volumes, including subnuclei, displayed comparable developmental trajectories between the very preterm and the full-term groups.
- Higher amygdala volumes at school-age were associated with higher emotional regulation difficulties in the very-preterm born group, and with lower affect recognition abilities in full-term born children and adolescents.
- In a subgroup of very-preterm children and adolescents followed from birth to school-age, no significant associations were found between amygdala volumes at term-equivalent age and socio-emotional competencies at school-age.

## Introduction

Preterm birth has long-lasting effects on neural architecture that increase the risk of developing neurodevelopmental and neuropsychiatric disorders over time. The fetal brain develops through a series of complex, time-critical events. Interruption to this specific process may lead to a cascade of impaired functioning mediated by both regional and global changes to brain architecture that can persist across life.^1–3^ After a very preterm birth (VPT; <32 weeks’ gestation), atypical brain development has long been categorized as a significant precursor to poor neurodevelopmental outcomes, with around 25% of VPT individuals experiencing behavioral problems, including socio-emotional difficulties that persist into adolescence and adulthood.^4–8^ Socio-emotional difficulties are observed as early as the first year of life in premature children and extend from difficulties in emotional information processing, emotion regulation, social-emotional understanding, socializing, peer relationships as well as internalizing problems.^8–11^ Importantly, deficits in these skills significantly increase vulnerability to psychiatric conditions and mental health problems.^12^ Of particular interest, amygdala abnormalities have been connected to poor socio-emotional outcomes in VPT children.^13^

Among brain regions vulnerable to prematurity, the amygdala holds a unique role. First, the amygdala is deeply involved in socio-emotional processing and has been associated with many of the socio-emotional and behavioral phenotypes common in preterm-born persons.^4^ Second, the amygdala holds a unique sensitivity to stress, which influences both amygdala size and connectivity.^14–20^ The prenatal and postnatal stress associated with prematurity, as well as the ability to mitigate this stress across life, pose important questions about the impact of premature birth on the developmental trajectory of the amygdala. While studies on preterm children and adolescents are scarce, existing literature has found consistently atypical amygdala volumes in preterm-born neonates and adults.^14,21–23^ Moreover, a recent study exploring amygdala subnuclei volume in 7-year-old VPT children showed that both lower right basal nuclei and greater central nuclei volumes were associated with socio-emotional problems.^13^ Atypical amygdala functional connectivity in VPT individuals has also been associated with socio-emotional impairment in VPT children^13,24^ and adults.^25,26^

While previous studies have showed atypical structural characteristics of the amygdala across various developmental stages in VPT infants, children, and adults, the developmental trajectory of this structure in comparison to full-term (FT) peers remains under explored. More particularly, there is a gap in understanding amygdala structural characteristics and their association with socio-emotional competencies during early adolescence. Our study aims to fill this gap using structural brain imaging data of preterm born children and adolescents spanning many years. Moreover, despite the recognized role of the amygdala structure in socio-emotional processing, research on this relationship has relied mainly on parental questionnaires in preterm-born children. Benefitting from brain structural imaging data of children and adolescents born preterm spanning multiple years and specific neuropsychological testing of socio-emotional competencies, our study aims to address this additional gap. In this work, we sought to understand the amygdala’s developmental trajectory in school-aged VPT individuals. Specifically, we aimed at exploring amygdala subnuclei volumes in VPT and full-term (FT) children and adolescents aged 6–14 years using cross-sectional data, as well as their associations with socio-emotional outcomes. This broad age range was selected as it aligns with the concept of “school-age years”^27^ and it allows for the exploration of  potential age-related effects in amygdala development at school-age. Additionally, secondary longitudinal analyses from newborn term-equivalent age (TEA) were completed on a subgroup of VPT participants. Amygdala growth rates from TEA to school-age were investigated using longitudinal data, as well as their association with socio-emotional competencies at school-age.

## Methods

### Participants

#### Primary cross-sectional analyses at school-age

Participants of the current study were recruited between January 2017 and July 2019 as part of the “Geneva Preterm Cohort Study”, at the age of 6–14 years (including two sub-studies completed in children and adolescents from 6 to 14 years of age, the ‘Mindful preterm teens’ study^28^; and ‘Vis-à-Vis’ study). 392 VPT children and adolescents born <32 gestational weeks between 01.01.2003 and 31.12.2012, in the Neonatal Unit at the Geneva University Hospital, Switzerland, and followed up at the Division of Child Development and Growth, were invited to participate. VPT children and adolescents were excluded if they had an intelligence quotient below 70, sensory or physical disabilities (cerebral palsy, blindness, hearing loss), or an insufficient understanding of French. A total of 108 VPT participants were enrolled. Of the 108 participants enrolled, 79 completed both the T1-weighted sequence of the brain magnetic resonance imaging (MRI) scan and neuropsychological assessment. A total of 75 VPT were included in the current study (out of the 79, *n* = 4 was discarded due to a high level of motion artefacts). Moreover, 46 FT children and adolescents aged between 6 and 14 years old were recruited through the community. Firstly, participants from the VPT group were encouraged to invite FT friends. Additionally, recruitment extended through informal channels, using word-of-mouth referrals from individuals affiliated with the Campus Biotech Geneva. Of the 46 participants, 41 completed both the T1-weighted sequence of the brain MRI scan and neuropsychological assessment. A total of 41 FT were included in the current study. All neuropsychological assessments and MRI acquisitions were completed at the University Hospital of Geneva or at the Campus Biotech in Geneva, Switzerland.

#### Secondary longitudinal analyses from TEA to school-age

Analyses were conducted on a “longitudinal” VPT subgroup. Of the 75 VPT included in the current study, 20 had an MRI at TEA with an image quality that allowed manual segmentation of the amygdala. Exclusion criteria included severe physical or sensory disabilities, chromosomal abnormalities, the presence of focal cerebral lesions, and bad image quality due to high level of motion artefacts on the T2-weighted image.

This study was approved by the Swiss Ethics Committees on research involving humans, ID: 2015-00175. Written informed consent was obtained from the principal caregiver and from the participant.

### Neonatal and demographic measures

Neonatal characteristics were documented from medical records. Socio-economic status (SES) of the parents was estimated using the Largo scale, a validated 12-point score based on maternal education and paternal occupation.^29^ Higher Largo scores reflect lower parental SES. General intellectual functioning was evaluated using two different measures according to the age of the participant. In participants from 6 years to 9 years and 11 months old, the Kaufman Assessment Battery for Children – 2nd Edition (K-ABC-II^30^) was used to evaluate the Fluid-Crystallized Index (FCI) as a measure of general intellectual functioning. In participants from 10 to 14 years of age, the Wechsler Intelligence Scale for Children – 5th Edition (WISC-V^31^) was used to evaluate the General Ability Index (GAI) as a measure of general intellectual functioning. The GAI is derived from the core verbal comprehension and perceptual reasoning subtests.^32,33^ Both measures of general intellectual functioning, FCI and GAI, have a mean of 100 and a standard deviation of 15. Further details are described in the Supplementary Methods.

### Socio-emotional measures

Participants’ socio-emotional competencies were assessed using four different measures, further details are given in the Supplementary Methods:

- Theory of Mind subtest of the Developmental Neuropsychological Assessment - 2nd Edition (NEPSY-II^34^): raw scores were regressed on age at testing and socio-economic status (SES evaluated using the Largo scale, see Supplementary Methods). Standardized residuals were used as a score, denoted hereafter as “theory of mind”. Higher scores reflect better theory of mind.
- Affect Recognition subtest of the NEPSY-II^34^: raw scores were regressed on age at testing and SES. Standardized residuals were used as a score, denoted hereafter as “affect recognition”. Higher scores reflect better affect recognition.
- Internalizing Problems Score of the Strength and Difficulties Questionnaire – parent version (SDQ^35,36^): the emotional symptoms subscale and the peer problems subscale were combined into a broadband internalizing problems score. Raw scores were regressed on age at testing and SES. Standardized residuals were used as a score, denoted hereafter as “internalizing problems”. High internalizing scores reflect higher internalizing difficulties in daily life.
- Emotional Control Scale of the Behavior Rating Inventory of Executive Function, parent version (BRIEF^37^): standardized scores were used (mean = 50, SD = 10) and regressed on SES. Standardized residuals were used as a score, denoted as emotional control. Higher emotional control scores reflect higher difficulties in “emotional control”. Higher emotional control scores reflect higher emotional control problems.

### Magnetic resonance imaging

#### MRI acquisition

In the context of the “Geneva Preterm Cohort Study”, structural T1-weighted MP-RAGE (magnetization-prepared rapid gradient-echo) sequences were acquired using the following parameters: voxel size = 0.9 × 0.9 × 0.9 mm; repetition time (TR) = 2300 ms; echo time (TE) = 2.32 ms; inversion time (TI) = 900 ms; flip angle (FA) = 8°; and field of view (FOV) = 240 mm.

For the MRI scan at TEA in the longitudinal subgroup, MRI data acquisition was performed without sedation during the infants’ natural sleep on three different MRI scanners due to scanner upgrades throughout the study period: Philips Intera (1.5 T), Philips Achieva (1.5 T), and Siemens Trio Tim (3 T). T2-weighted images were acquired using similar sequences across the different scanners: turbo spin-echo sequence (TSE), TE = 150 ms, TR = 4600 ms, 113 coronal slices, voxel size: 0.8 × 0.8 × 1.2 mm.

Further details of MRI acquisition are given in the Supplementary Methods.

#### Volumetric measures

**Fig. 1:**
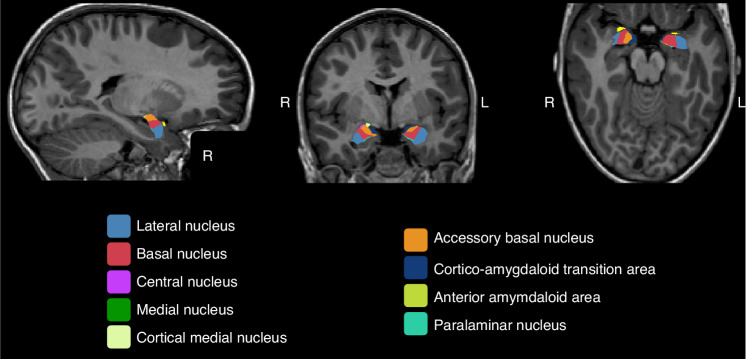
Illustration of amygdala subfield segmentation by FreeSurfer (version 7.1.1). Amygdala subfields are shown from left to right in sagittal, coronal and axial views, respectively.

T1-weighted images from the “Geneva Preterm Cohort Study”, were segmented using the recon-all function from Freesurfer software (version 7.1.1). The amygdala nuclei module was used to produce an automated segmentation of 9 bilateral amygdala nuclei (i.e., anterior amygdaloid area, cortico-amygdaloid transition area; basal, lateral, accessory basal, central, cortical medial, paralaminar nuclei; see Fig. 1 for illustration), as well as the total volume for the left and the right amygdala.

Previous literature suggests that FreeSurfer version 6 and above is generally a reliable segmentation tool for segmentation and volume extraction for amygdala nuclei.^38^ The quality control process used in this study followed similar guidelines to those detailed by Backhausen et al.^39^ First, the subfield and nuclei segmentations were manually inspected for obvious errors. Second, volumes were extracted twice for each participant and datasets were compared for any disparities. Volumes that exceeded 5 standard-deviation from the mean of each region studied were excluded.

**Fig. 2:**
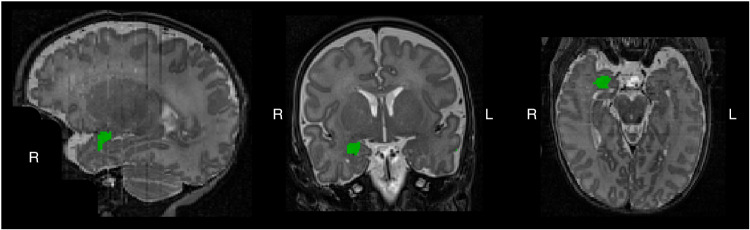
Illustration of the right amygdala manual delineation using ITK-SNAP software, (version 3.6.0). The right amygdala, in green, is shown from left to right in sagittal, coronal and axial views, respectively.

For TEA data, segmentation of the whole amygdaloid complex was performed on T2-weighted images by a physician trained in this task for six months. The anatomical delineation was based on guidelines for the localization of the amygdala, see Fig. 2 for illustration.^40,41^ Neonatal amygdala volumes were manually traced for each subject using ITK-SNAP software. Neonatal amygdala volumes corresponding to the manual segmentation was calculated automatically by the ITK-SNAP software and expressed in cubic millimeters. To improve segmentation quality and reduce errors associated with manual segmentation imprecision, the left and right amygdala were traced five times for each participant by the same delineator and using the same methodology for all scans. The individual’s median amygdala volume was calculated for the five drawings and was used for further analysis. Detailed manual segmentation methodology is described in the Supplementary Methods. ICV was segmented using the automatic segmentation method of Gui et al.^42^

### Statistical analyses

#### Demographic and socio-emotional competencies group-wise comparison

Differences between groups in demographic and socio-emotional measures were analyzed using independent-sample t-tests. P-values were corrected for multiple comparisons with the false discovery rate (FDR^43^).

#### Primary cross-sectional analyses at school-age

##### Group-wise comparison of amygdala volumes at 6–14 years - ANCOVA analysis

Differences between groups in amygdala nuclei and total volumes were analyzed using multivariate analysis of covariance (ANCOVA), including adjustment for ICV, sex and SES (i.e., Largo score). To compare volumetric maturation over time between the VPT and FT groups, an interaction effect of group x age was added to the model. For the 20 ANCOVA models (one for each amygdala volumetric measure), p-values were corrected for multiple comparisons with the false discovery rate (FDR^43^). Analyses were performed using the R software version 4.0.3, and R studio version 1.3.1093.^44,45^

##### Multivariate relationships between raw amygdala volumes and socio-emotional measures at 6–14 years

To evaluate multivariate patterns of association between amygdala volumetric measures and socio-emotional competencies, we applied a partial least squares correlation (PLSC) using a publicly available PLSC implementation in MATLAB: https://github.com/danizoeller/myPLS.^46,47^ PLSC is a data-driven multivariate technique that maximizes the covariance between two matrices by identifying latent components which are linear combinations of the two matrices, i.e., raw amygdala volumetric measures and socio-emotional measures.^48^

In short, a cross-covariance matrix (R) is computed between the raw amygdala volumetric measures and the socio-emotional measures, i.e., affect recognition, theory of mind, internalizing problems, and emotional control for each group, which were then concatenated. Singular value decomposition was then applied to this cross-covariance matrix (R), resulting in latent components. Each latent component is composed of a set of socio-emotional weights (different for each group) and amygdala volume weights (common to all participants), which indicate how strongly each socio-emotional and raw amygdala volume variable contribute to the multivariate socio-emotional/amygdala volume association. Therefore, each group has its own socio-emotional pattern associated to a common amygdala volume pattern across both the VPT and FT groups. Statistical significance of latent components was assessed with permutation testing (1000 permutations) and considered robust at *p* < 0.01 following guidelines of previous studies.^46,47^ Stability of saliences were estimated using bootstrapping (500 bootstrap samples with replacement). Bootstrap ratio z-scores for raw amygdala volumetric measures and socio-emotional measures were obtained by dividing each amygdala volumetric and socio-emotional weight by its bootstrap-estimated standard deviation, and a p-value was obtained for each bootstrap ratio z-score. The contribution of amygdala volumetric and socio-emotional weights for a given latent component was considered robust at *p* < 0.01 (i.e., absolute bootstrap ratio z-scores above 3 or below −3).

#### Secondary longitudinal analyses from TEA to school-age

##### Qualitative analysis of longitudinal trajectories of amygdala volumes in the VPT subgroup

Amygdala volume relative growth rates from TEA to school-age was calculated for the total volume as well as left and right amygdala volumes separately, using the following formula:

$$	{Amygdala\; Relative\; Growth\; Rate}\,( \% /{month}) \\ 	 =\left[\frac{{Raw\; amygdala\; vol}\left({school\; age}\right)-{Raw\;amygdala\; vol}\left({TEA}\right)}{{{{{{\rm{Age}}}}}}\; {{{{{\rm{in}}}}}}\; {{{{{\rm{months}}}}}}({{{{{\rm{school}}}}}}\; {{{{{\rm{age}}}}}})-{{{{{\rm{Age}}}}}}\; {{{{{\rm{in}}}}}}\; {{{{{\rm{months}}}}}}({{{{{\rm{TEA}}}}}})}\right]\times \frac{100}{{Vol}({TEA})}$$

Three plots were extracted to illustrate the growth rate of the total, right and left amygdala volumes from TEA to school-age. Additional plots presenting raw amygdala volume and ICV-corrected amygdala volumes in relation to age at MRI were also extracted.

*Multivariate relationships between raw amygdala volumes at TEA and socio-emotional measures at school-age; and between longitudinal amygdala volume trajectories (from TEA to school-age) and socio-emotional measures:*

A second PLSC was used to evaluate associations between raw amygdala volumes at TEA and socio-emotional measures at school-age. A procedure similar to the previous PLSC was employed using a 20 × 2 matrix denoted X containing raw left and right amygdala volumes at TEA and a 20 × 4 matrix denoted Y containing the 4 socio-emotional measures, i.e., affect recognition, theory of mind, internalizing problems and emotional control.

A third PLSC was used to evaluate associations between longitudinal amygdala volume trajectories and socio-emotional measures at school-age. A qualitative examination of Fig. 4, illustrating the individual longitudinal growth of the total amygdala volume from TEA to school-age, reveals a gradual decline in the slope of the lines as the children’s age increases. This phenomenon may be attributed in part to the extended time between the scans conducted at TEA and school-age for older children, with the duration increasing as the child ages and resulting in a reduction in the slope. Therefore, and based on previous studies,^49^ for longitudinal amygdala volume trajectories, the delta for left and right raw amygdala volume was calculated for each participant, i.e., Δ_Volume_ = raw amygdala volume at school-age – raw amygdala volume at TEA. Given the presence of two time points per subject with various age at assessment at school-age and to correctly capture the within-subject effect of age on amygdala volumes developmental trajectory, we used the inter age previously defined as the interaction between mean age and delta age (i.e., Δ_Age_ = age at assessment at school-age – age at assessment at TEA), which enables the capture of convex (i.e., U-shaped) neurodevelopmental trajectories.^49^ Finally, Δ_Volume_ were regressed on the inter age previously defined. Standardized residuals were used as a score of developmental amygdala volume trajectories. A similar PLSC procedure was employed using a 20 × 2 matrix denoted X containing left and right-lateralized scores of developmental amygdala volume trajectories and a 20 × 4 matrix denoted Y containing the 4 socio-emotional measures, i.e., affect recognition, theory of mind, internalizing problems, and emotional control.

## Results

### Participant characteristics and socio-emotional competencies

#### Primary cross-sectional analyses at school-age

The final sample included 75 VPT and 41 full-term participants between 6 and 14 years of age. Neonatal and demographic characteristics are shown in Table 1. Within this cohort, none of the participants exhibit severe brain injury.

**Table 1:** Neonatal, demographic characteristics and socio-emotional competencies of VPT and FT participants from the cross-sectional study.

	VPT	FT	Group comparison
Neonatal characteristics			
Gestational Age, in weeks (mean (SD))	29.42 (1.84)	39.9 (1.34)	*t*(114) = 32.077, *p* < 0.001
Birth weight, in grams (mean (SD))	1260.93 (368.21)	3460.98 (413.8)	*t*(114) = 29.435, *p* < 0.001
IVH—Grades III and IV, *n* (%)	3 (4%)	0 (0%)	–
Cystic periventricular leukomalacia	1 (1.3%)	0 (0%)	–
Demographic characteristics			
Age at assessment, in months (mean (SD))	124.07 (27.68)	123.68 (26.57)	*t*(114) = 0.072, *p* = 0.942
Sex: - Female, *n* (%) - Male, *n* (%)	39 (52%)	17 (41.46%)	X^2^(1) = 1.179, *p* = 0.278
	36 (48%)	24 (58.54%)	
SES, Largo score (mean (SD))	4.24 (2.32)	2.89 (1.29)	*t*(110) = −3.325, *p* = 0.001
General intellectual functioning (mean (SD))	106.58 (12.6)	113.88 (10.95)	*t*(113) = 3.113, *p* = 0.002
Socio-emotional competencies			
Theory of Mind (mean (SD))	−0.01 (0.92)	0.02 (1.16)	*t*(65.68) = −0.180, *p* = 0.858, *q* = 0.858, *d* = 0.0379
Affect Recognition (mean (SD))	−0.07 (1.06)	0.13 (0.90)	*t*(91.91) = −1.0808, *p* = 0.2826, *q* = 0.377, *d* = 0.201
Internalizing Problems (mean (SD))	0.19 (1.04)	−0.37 (0.83)	*t*(96.43) = 3.152, *p* = 0.002, *q* = 0.009, *d* = 0.576
Emotional Control (mean (SD))	0.15 (1.01)	0.15 (1.01)	*t*(85.72) = 2.258, *p* = 0.026, *q* = 0.053, *d* = 0.431

Notes: For socio-emotional competencies: (a) standardized residuals, corrected for age and socio-economic status, are used as a score as described in the methods, (b) *q*-value, corrected *p*-values using FDR, (c) effect sizes were calculated using Cohen’s d for two-sample *t*-test; IVH Intraventricular hemorrhage.

Characteristics were similar between the VPT and FT groups for sex and age at assessment. SES, as measured by the Largo scale, showed significant group difference, with lower SES (i.e., higher Largo score) in the VPT group compared to the FT group. Similarly, general intellectual functioning was in the average range for both groups but showing significant group difference with reduced general intellectual functioning in the VPT compared to the FT group. Socio-emotional competencies were comparable between the two groups, except for the Internalizing Problems with significantly higher problems in the VPT group compared to the FT group, Table 1.

#### Secondary longitudinal analyses from TEA to school-age

Amygdala volumes at TEA were available for 20 VPT participants from the previous cross-sectional analyses. None of the participant from the VPT subgroup show IVH - Grades III and IV. Neonatal and demographic characteristics as well as socio-emotional competencies of the longitudinal VPT participants are presented in Table 2. Within this cohort, none of the participants exhibit additional forms of brain injury.

**Table 2:** Neonatal, demographic characteristics and socio-emotional competencies of VPT from the longitudinal subgroup.

	VPT subgroup
Neonatal characteristics	
Gestational Age, in weeks (mean (SD))	28.60 (1.78)
Birth weight, in grams (mean (SD))	1100.75 (407.31)
IVH—Grades III and IV, *n* (%)	0 (0%)
Cystic periventricular leukomalacia	0 (0%)
Demographic characteristics at school-age	
Age at assessment at school-age, in months (mean (SD))	122.15 (31.73)
Sex: - Female, *n* (%)	10 (50%)
- Male, *n* (%)	10 (50%)
SES, Largo score (mean (SD))	3.6 (1.76)
General intellectual functioning (mean (SD))	110.15 (9.04)
Socio-emotional competencies at school-age	
Theory of Mind (mean (SD))	0.199 (0.93)
Affect Recognition (mean (SD))	−0.178 (1.03)
Internalizing Problems (mean (SD))	−0.004 (0.81)
Emotional Control (mean (SD))	0.148 (1.25)

Notes: IVH Intraventricular hemorrhage.

### Primary cross-sectional analyses at school-age

#### Group-wise comparison of amygdala volumes

The VPT and the FT groups were compared for each amygdala subnuclei volume, as well as for developmental volumetric maturation over time. The results showed no significant group difference, or group x age difference, with a small effect size overall. Considering ANCOVA analyses, the adjustment for ICV, sex and SES showed a significant impact of ICV for all amygdala volumes; a significant impact of sex for 4 of the left-lateralized amygdala subnuclei, i.e., basal nucleus, accessory basal nucleus, central nucleus and paralaminar nuclei, as well as for the left total amygdala volume with higher amygdala volume in male compared to female; and no significant contribution of SES. See Supplementary Table S1 and Supplementary Figs. S1 and S2.

#### Association between amygdala volumes and socio-emotional measures at school-age

PLSC analysis applied to amygdala volumes and socio-emotional measures resulted in one significant component, i.e., latent component 1 (LC1), *p* = 0.032.

**Fig. 3:**
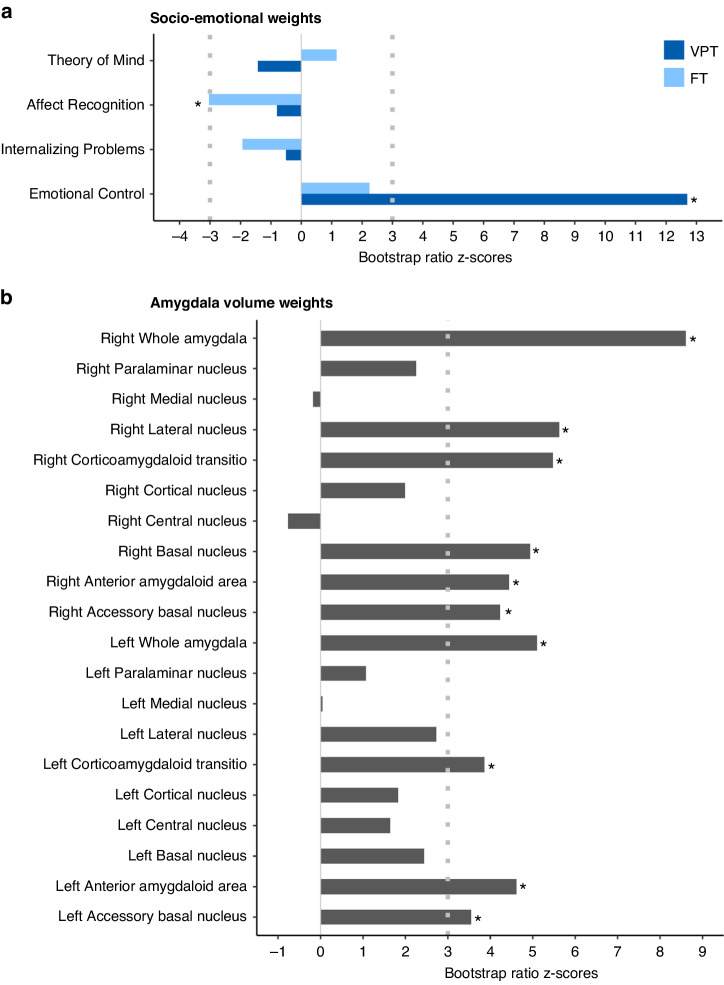
Associations between amygdala volumetric measures and socio-emotional competencies at school-age in the very preterm (VPT) and full-term (FT) groups based on the PLSC analysis. Each latent component is composed of a set of socio-emotional weights (different for each group) and amygdala volume weights (common to all participants), which indicate how strongly each socio-emotional and amygdala volume variable contribute to the multivariate socio-emotional/amygdala volume association. **a** The diverging graph shows socio-emotional bootstrap ratio z-scores for the VPT and the FT groups in dark blue and in light blue, respectively. Socio-emotional measures with an absolute bootstrap ratio z-score ≥ 3 or ≤−3 yield a robust contribution to the component and are marked with a star. **b** The diverging graph shows amygdala volume bootstrap ratio z-scores. Amygdala volumes with an absolute bootstrap ratio z-score ≥ 3 or ≤−3 yield a robust contribution to the component and are marked with a star.

In the VPT group, LC1 revealed a significant association between amygdala volumetric measures and higher emotional regulation difficulty in everyday life. In the FT group, higher amygdala volumes were associated with reduced affect recognition scores. Overall, for both groups, decreased amygdala volumes were associated with better socio-emotional competencies, more specifically in the accessory basal, anterior amygdaloid area, cortico-amygdaloid transition area bilaterally and at the whole amygdala; as well as in the right lateral, basal, and cortical medial subnuclei. Figure 3 shows the saliences of each amygdala volumetric and socio-emotional measures for LC1. Original saliences, as well as their bootstrap-estimated standard deviations and bootstrap ratio z-scores for the PLSC analyses showing a significant latent component are reported in Supplementary Table S2.

Additional analyses were conducted to evaluate the potential role of gestational age in the association between amygdala volumes and socio-emotional measures at school-age. In this regard, socio-emotional scores were regressed out on gestational age, in addition to age at testing and socio-economic status already outlined in the methods section. The revised socio-emotional scores were then used in the PLSC analysis. Application of PLSC to amygdala volumes and socio-emotional measures adjusted for gestational age revealed a significant component, specifically latent component 1 (LC1), *p* = 0.024. The observed patterns of association were highly comparable to the results obtained without considering gestational age, see Supplementary Table S3 and Supplementary Fig. S3.

### Secondary longitudinal analyses from TEA to school-age

#### Amygdala longitudinal amygdala growth from TEA to school-age

**Fig. 4:**
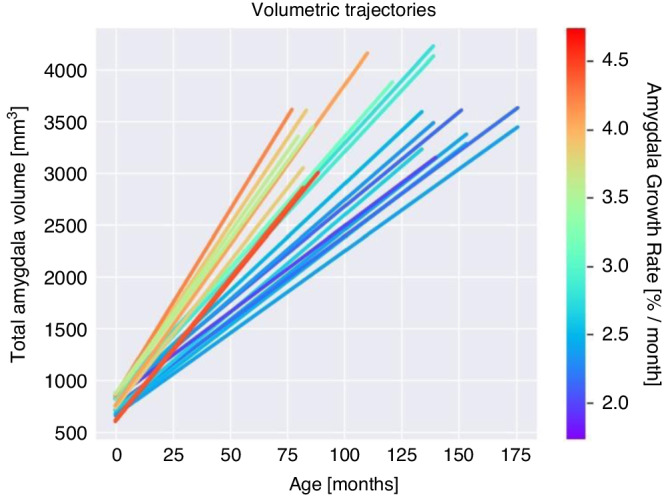
Line plot showing individual longitudinal growth of the total amygdala volume from TEA to school-age. x-axis: age in months; y-axis: total amygdala volume in mm3. Line colors indicate amygdala growth rates relative to the amygdala volume at TEA (faster and slower growth are represented by red and blue colors, respectively).

The relative growth rate of the amygdala was calculated for each participant. Using a qualitative observation of Fig. 4 of the total amygdala volume (and Supplementary Fig. S4 for left and right amygdala), the slope of the lines gradually decreases as the age of the children increases. Faster growth (red and orange lines) is found in children aged between 6 to 9 years old; while slower growth (blue and violet lines) is found in older children. Left and right relative growth rate of the amygdala are shown in Supplementary Fig. S3. Raw amygdala volume and ICV-corrected amygdala volume in relation are also shown in Supplementary Figs. S5 and S6.

#### Association between amygdala volumes at TEA with socio-emotional measures at school-age, and between longitudinal amygdala volumetric trajectories with socio-emotional measures at school-age

In the subgroup of 20 VPT participants, PLSC analyses revealed no significant latent component. Therefore, there was no significant association between amygdala volumes at TEA and socio-emotional competencies at school-age (LC1, *p* = 0.475), Supplementary Table S4; neither between longitudinal amygdala volume trajectories and socio-emotional competencies at school-age (LC1, *p* = 0.387), Supplementary Table S5.

Additional analyses were conducted to evaluate the potential role of gestational age in the association between amygdala volumes at TEA with socio-emotional measures at school-age, and between longitudinal amygdala volumetric trajectories with socio-emotional measures at school-age. In this regard, socio-emotional scores were regressed out on gestational age, in addition to age at testing and socio-economic status already outlined in the methods section. The revised socio-emotional scores were then used in the two previously described PLSC analysis. Application of PLSC to amygdala volumes at TEA and socio-emotional competencies adjusted for gestational age at school-age (LC1, *p* = 0.210) and to longitudinal amygdala volume trajectories and socio-emotional competencies adjusted for gestational age at school-age (LC1, *p* = 0.228) revealed no significant latent component. The observed patterns of association were highly comparable to the results obtained without considering gestational age, see Supplementary Table S6 and Supplementary Table S7.

## Discussion

In this study, we have used the amygdala’s volumetric measures across different ages to explore its developmental trajectory across infancy, childhood and adolescence, and related socio-emotional competencies after a VPT birth. Considering cross-sectional data in children and adolescents 6–14 years of age, the VPT and their FT counterparts showed comparable volumetric characteristics as well as comparable developmental volumetric maturation over time at the whole-amygdala and at the subnuclei level. Our study revealed relationships between amygdala volumetric measures and socio-emotional outcomes at school-age, with greater amygdala volumes related to more emotional regulation difficulties in VPT participants and lower affect recognition competence in FT participants. Considering secondary longitudinal data from TEA to school-age, we observed that the amygdala’s growth rates (from TEA to childhood or adolescence) were higher in children aged between 6 and 9 years old than in young adolescents (10–14 years old). At TEA, amygdala volumes showed no associations with socio-emotional scores at school-age. Furthermore, there was also no association between amygdala volumetric developmental trajectory from TEA to school-age with socio-emotional scores at school-age. Our results help paint a picture of the complexity in studying amygdala development in VPT infants, children and adolescents. Below, we explore in further detail both the nature and significance of our results.

### VPT and FT groups showed comparable amygdala volumetric characteristics at school-age

Using cross-sectional data, we found no significant differences in amygdala volumetric measures at both the whole-amygdala level and at the nuclei level between VPT and FT children and adolescents. While the exploration of amygdala volume is scarce in preterm children, especially at school age, our results contrast general findings that show a reduction of amygdala volumes compared to FT controls. At TEA, Cismaru and colleagues (2016) found reduced amygdala sizes in VPT-born infants compared to their FT peers.^50^ Similar reduction of amygdala volumes has been observed in preterm children,^51^ adolescents,^52,53^ and adults.^21,54–56^ In VPT adults, a recent meta-analysis aligns with the premise of lower amygdala volumes following VPT birth, while also noting the presence of variation in effect sizes among studies examining amygdala volume.^57^ For VPT children, changes in amygdala size and connectivity seem to be influenced by many variables, including postnatal and prenatal factors. Indeed, a recent study by Chau et al. suggests that neonatal clinical factors and genotypes account for 46% of the overall variance in amygdala volume in VPT children at school-age.^58^ The authors also found that lower amygdala volumes are significantly associated with greater neonatal invasive procedures (i.e., exposure to pain-related stress) in VPT children. This may suggest that our preterm group has experienced resilience-increasing factors. Consistent with this hypothesis, our cohort included mostly well-functioning VPT children and adolescents that present with amygdala volumetric maturation comparable to FT controls. Indeed, our cohort shows general intellectual abilities mostly within the average to high average range (IAG mean (SD) = 106 (12.6)) while previous studies conducted in children showed full-scale intellectual quotient (FSIQ) in the average to low average range (mean FSIQ between 80 and 99).^52,53^

### The amygdala’s developmental trajectory after very preterm birth: from TEA to school-age

Using longitudinal data in a VPT subgroup and through qualitative investigations of the amygdala volumetric growth rate from TEA to school age, we observed that the slope of the growth rate from birth to age 6–9 is steeper compared to a broader timeframe spanning birth to age 10–14. This suggests a potentially higher growth rate during childhood than adolescence. However, this may also be influenced by a shorter time period between TEA and the 6–9 years scans, compared with the time span between TEA and the 10–14 years scans. Nevertheless, these findings align with existing literature on typically developing individuals, as described by Uematsu and colleagues (2012), which found initial rapid amygdala growth in early childhood followed by a plateau in late childhood and early adolescence.^59,60^ Indeed, in typically developing individuals, the study of Uematsu and colleagues showed a non-linear age-related amygdala volume maturation with cubic models best characterizing the estimated developmental trajectories.^60^

Literature also shows that timing plays an important role in both typical and atypical amygdala development, showing sensitive periods with higher growth rates, especially during early childhood.^61^ Other factors, like sex, lateralization, and external factors (maternal closeness, defined as both physical and emotional closeness between the caregiver and the child) also impact rates of growth in the amygdala.^61^ A study by Russell et al. found that 83% of the variance in amygdala growth rates in their cohort of typically developing children was due to between-person differences, like pubertal stage or stress.^62^ Even though these children had not experienced any periods of abnormally acute or prolonged stress, their amygdalar growth still varied significantly, suggesting that even small stressors, aggregated to other environmental and biological factors, produce variations in developmental trends within subjects.

### Socio-emotional outcomes at school-age are related to amygdala volume at school-age but not at TEA

When exploring association with socio-emotional outcomes at school-age, our findings show significant association with current amygdala volumetric measures (cross-sectional data), but not with amygdala volumes at TEA, nor with longitudinal amygdala volumetric trajectories from TEA to school-age (longitudinal data).

Using cross-sectional amygdala volumetric measures in VPT and FT children and adolescents, greater volumes in the accessory basal, anterior amygdaloid area, cortico-amygdaloid transition area of both the left and right amygdala; as well as the whole amygdala and right lateral, basal, and cortical medial nuclei, were associated overall with deficits in different socio-emotional competencies in both the VPT and FT groups. More specifically, in VPT children and adolescents, higher amygdala volumes were associated with lower emotional regulation competencies; while in FT children and adolescents, higher amygdala volumes were associated with lower affect recognition competencies. These findings are coherent with a study by Cismaru et al., which showed an association between larger amygdala volume at TEA and a pronounced fear escape reaction versus blunted fear reaction with lower amygdala volumes at 12 months.^50^ Furthermore, a recent investigation involving 7-year-old VPT children revealed associations between amygdala subnuclei volume in the right basal nuclei and central nuclei, and greater social problems as rated by parents using the Child Youth and Behavioral Checklist (CBCL) in the social problem subscale.^13^ This partly aligns with our findings, as greater right central nuclei volume was linked to greater socio-emotional problems, while lower right basal nuclei volumes was similarly associated with higher socio-emotional competencies. Several studies have found larger amygdala sizes in children to be linked to more pronounced social and communication difficulties, especially in the context of autism spectrum disorder (ASD).^63,64^ Similarly, in a longitudinal study of adolescents with ASD, higher amygdala volume was associated with greater ASD symptomatology.^65^ Numerous socio-emotional challenges observed in children with ASD share commonalities with socio-emotional difficulties in preterm-born children, encompassing issues related to emotional regulation, affect recognition, and theory of mind. This overlap has been emphasized by Johnson and Marlow (2011) reporting a 3- to 4-fold increased risk of ASD, the inattentive subtype of attentional deficit hyperactivity disorder (ADHD), and anxiety disorders in VPT children.^66^ Within the context of ASD, this extends beyond an increased susceptibility to disorders and includes subclinical symptoms related to socio-emotional difficulties. The convergence of socio-emotional profiles also suggests that atypical morphometry of the amygdala could impact specific features of socio-emotional functioning in preterm children.

Using longitudinal amygdala volumetric characteristics from TEA to school-age, we found no significant association of socio-emotional outcomes at school-age neither with amygdala volume at TEA, nor with longitudinal amygdala volumetric trajectories from TEA to school-age. These findings contrast with studies that predominantly show an association at a young age. In preterm children, amygdala volumes at TEA have been previously associated with fear-processing reactions at 12 months^50^ as well as with emotional symptoms at 5-years-old.^67^ These discrepancies could be due in part to the impact of neuro-plastic responses occurring during childhood or to different methodological approaches used to evaluate the association between volumetric data at TEA and socio-emotional outcomes.

### Limitations

One of the major limitations of the current study is the limited number of patients with longitudinal amygdala data. Moreover, we did not have a FT control group for the longitudinal subgroup of VPT children in the current study. This limited the longitudinal analyses and trajectories that we could establish. Finally, the broad age range coupled with limited sample size used in this study might, both in the cross-sectional and longitudinal analyses, limit our ability to capture developmental changes and dynamics occurring in both brain structure and socio-emotional functioning, as well as its interaction. Nevertheless, and based on previous studies, we believe that comparable amygdala and social-emotional development between the two groups is more likely due to well-functioning individuals in the VPT group who exhibit a functioning profile akin to their FT peers in terms of both amygdala and socio-emotional development. Research with more inter-individual variability in the VPT population might be needed to paint a more comprehensive picture of the architecture of a VPT child’s amygdala development in infancy, childhood and adolescence.

## Conclusion

Cross-sectional findings showed comparable amygdala volumetric characteristics and developmental trajectory between the VPT and the FT groups, contrary to general findings of atypical amygdala volumes in VPT individuals. In our cohort, predominantly composed of well-functioning VPT children and adolescents, potential resilience-enhancing factors might have played a role in these positive outcomes. In both groups, similar increased in amygdala volumes were associated with socio-emotional difficulties. These associations highlight the role of amygdala structural characteristics on socio-emotional functioning across populations. In the longitudinal VPT subgroup, the qualitative observation of amygdala developmental trajectories from TEA to school-age seem comparable to studies conducted in typically developing children.

In conclusion, based on the literature, there is a clear need to support VPT individuals who experience long-term socio-emotional deficits due to their prematurity, as these deficits can compromise their social and emotional trajectory. As childhood and adolescence are an appropriate period for intervention, for example through school-based interventions, developing a greater understanding of the links between brain atypicality and socio-emotional development could allow for more targeted and effective interventions to enhanced socio-emotional competencies in VPT individuals.^68,69^

## Supplementary information

Supplementary information

## Data Availability

Deidentified individual participant data will be made available upon publication to researchers who provide a methodological proposal for use in achieving the goals of the approved proposal. Proposals should be submitted to Petra.Huppi@unige.ch.
