# Supplementary material for: Amygdala volumes and associations with socio-emotional competencies in preterm youth: cross-sectional and longitudinal data
Source: Pediatr Res. 2024 May 18;96(7):1868–77. doi: 10.1038/s41390-024-03227-y (PMC11772232; doi:10.1038/s41390-024-03227-y)
Supplement: Supplementary file 1 — Supplementary information [file 41390_2024_3227_MOESM1_ESM.pdf]

Supplementary Materials

Supplementary Methods

Neonatal and Demographic measures

Neonatal characteristics were documented from medical records. Socio-economic status (SES) of the parents was estimated using the Largo scale, a validated 12-point score based on maternal education and paternal occupation (1). Higher Largo scores reflect lower SES of the parents. General intellectual functioning was evaluated using two different measures according to the age of the participant. In participants from 6 years to 9 years and 11 months old, the Kaufman Assessment Battery for Children – 2nd Edition (K-ABC-II; (2)) was used to evaluate the Fluid-Crystallized Index (FCI) as a measure of general intellectual functioning. The FCI is derived from a linear combination of 10 core subtests that composed five first-order scale scores (i.e., Short-Term memory, Long-Term Storage and Retrieval, Visual Processing, Fluid Reasoning, and Crystallized Ability). For children younger than 7 years of age, a different subset combination is administered to calculate the FCI. In participants from 10 to 14 years of age, the Wechsler Intelligence Scale for Children – 5th Edition (WISC-V; (3)) was used to evaluate the General ability index (GAI) as a measure of general intellectual functioning. The GAI is derived from the core verbal comprehension and perceptual reasoning subtests. Both of these measures of general intellectual functioning, FCI and GAI, have a mean of 100 and a standard deviation of 15.

Socio-emotional measures

• Theory of Mind subtest of the Developmental Neuropsychological Assessment - 2nd Edition (NEPSY-II; (4)):

The theory of mind subtest measures understanding of mental functions and other people's perspectives. In the first task, questions are asked to the participant about different verbal scenarios measuring understanding of beliefs, intentions, others' thoughts, ideas and comprehension of figurative language. In the second task, participants have to match facial emotional expressions, from photographs of children's faces, to a scenario. Raw scores were regressed on age at testing

and socio-economic status (SES evaluated using the Largo scale, see supplementary methods). Standardized residuals were used as a score, called “theory of mind”. Higher scores reflect better theory of mind abilities.

• Affect Recognition subtest of the NEPSY-II (4)

The affect recognition subtest assesses the ability to recognise facial emotional expressions (happy, sad, anger, fear, disgust, and neutral) from photographs of children’s faces in several matching tasks. In the first task, the participant selected one of the four faces that depicted the same emotion as a child's face at the top of the page. In a second task, the participant selected two photographs of faces that displayed the same affect from a selection of four photographs. Finally, the participant examined a photograph of a child's face for 5 seconds, and then from memory, selected two photographs that matched the same emotion as the face previously shown. Raw scores were regressed on age at testing and SES. Standardized residuals were used as a score, called “affect recognition”. Higher scores reflect better affect recognition abilities.

• Internalized Score subscale of the Strength and Difficulties Questionnaire – parent version (SDQ; (5,6)):

The SDQ parent questionnaire assess overall behaviour problems, emotional symptoms, hyperactivity and inattention, peer relationship problems, and prosocial behaviour. It rates participant's behaviour over the previous 6 months. The SDQ is scored on a Likert scale and includes 25 items, providing a Total Difficulties score, as well as an internalised and externalised score. In the current study, given our interest in socio-emotional outcomes, we focused on the internalized score. Raw scores were regressed on age at testing and SES. Standardized residuals were used as a score, called” internalized problems”. High internalized scores reflect increased internalized difficulties in daily life. Higher internalized scores reflect increased internalized problems.

• Emotional Control Scale of the Behavior Rating Inventory of Executive Function, parent version (BRIEF; (7)):

The BRIEF parent questionnaire provides an index of attention, hyperactivity and impulsivity in everyday life. The BRIEF comprises 86 items over two standardised subscales: (i) Behavioural Regulation Index (BRI) comprising 3 subscores including, Inhibit, Shift, Emotional Control; (ii) Metacognition index (MI) comprising 5 subscores including, Initiate, Working Memory, Plan/Organise, Organisation of Materials, Monitor; as well as a global score called the Global Executive Composite (GEC). In the current study, given our interest in socio-emotional outcomes, we focused on the emotional control score. Standardized

scores were used (mean = 50, SD = 10) and regressed on SES. Standardized residuals were used as a score, called emotional control. Higher emotional control scores reflect increased difficulties in "emotional control". Higher emotional control scores reflect increased emotional control problems.

MRI acquisition

Primary analyses: In the context of the "Geneva Preterm Cohort Study", MRI data were acquired at the Campus Biotech in Geneva, Switzerland, using a Siemens 3T Magnetom Prisma scanner. All participants completed a simulated "mock" MRI session prior to their MRI scan. This preparation process was conducted by trained research staff and allowed participants to familiarize themselves with the scanner and the scanning process, eventually raising any concerns they might have had prior to the MRI scan. Furthermore, this process is known to facilitated acquisition of good quality MRI images in children and adolescents.(8,9) Structural T1-weighted MP-RAGE (magnetization-prepared rapid gradient-echo) sequences was acquired using the following parameters: voxel size = 0.9 × 0.9 × 0.9 mm; repetition time (TR) = 2,300 ms; echo time (TE) = 2.32 ms; inversion time (TI) = 900 ms; flip angle (FA) = 8°; and field of view (Fov) = 240 mm.

Secondary analyses: For the MRI scan completed at TEA in the longitudinal subgroup, MRI data acquisition were performed without sedation during the infants' natural sleep. Infants were positioned inside the scanner, wrapped in a vacuum pillow, and monitored with electrocardiography and pulse oximetry. In addition, earmuffs were used for noise attenuation. Imaging data were acquired at the Geneva University Hospital (HUG) using tree different MRI scanners due to scanner upgrades throughout the study period: Philips Intera (1.5T), Philips Achieva (1.5T), and Siemens Trio Tim (3T). Neonatal brain contains immature structures with different tissue composition than adults and the preferred structural image sequence at TEA are T2-weighted scans. T2-weighted images were acquired using similar sequences across the different scanner: turbo spin-echo sequence (TSE), TE = 150 ms, TR = 4600 ms, 113 coronal slices, voxel size: 0.8 x 0.8 x1.2 mm. Adequate signal-to-noise ratio (SNR) and absence of geometric distortions were verified on each scanner using regular phantom-based quality control programs provided by the vendors. To obtain comparable images, scanning protocols were harmonized among the machines during the study period. Since infant cortex can be as thin as 1-2 mm in some areas, we used the smallest slice thickness possible in order to capture and allow the segmentation of such fine anatomical details (1.5 mm for the older scanners, and 1.2 mm for the more recent scanners). In-plane resolution was set so as to obtain similar voxel volumes, and thus

similar SNR among scanners, namely to 0.7 mm x 0.7 mm on the older scanners, and 0.8 mm x 0.8 mm on the more recent scanners, yielding voxel volumes of 0.735 mm3, and 0.768 mm3 respectively. To obtain similar contrast on all scanners, the values of echo times, repetition times, and echo train lengths were chosen to be as close as possible among scanners. This method was previously described in Gui and colleagues (10).

Volumetric measures – Secondary analyses - Manual segmentation of the amygdala at TEA:

The anatomical delineation was based on guidelines for the localization of the amygdala (11). Bayer and Altman's histological atlases were used to validate and correct anatomical landmarks (12). For all scans, amygdala segmentations were performed using the same delineator and the same methodology: tracing began in the coronal plane to define the rostral pole of amygdala by the appearance of the basolateral nuclei. This was based on visual inspection as the most rostral slice on which nuclear grey matter can be seen subjacent to the cortex. Then, the amygdala was traced in sequential slices moving from rostral to caudal on the coronal slice. Moving one slice caudally from the appearance of the basolateral nuclei, the dorsomedial boundary of the amygdala become the cerebrospinal fluid (CSF). The semiannular sulcus (SAS) was used to separate the entorhinal cortex from the amygdala. Then, moving caudally the superior boundary was traced until it could no longer be seen as a distinct nucleus. At this point putamen and pallidum were used to delimit amygdala's superior border. The dorsolateral and ventrolateral boundaries were defined by white matter (external capsule and anterior commissure) and/or CSF from the temporal horn. Moving caudally, the hippocampal formation was used as the ventral border. After delineation on coronal slices, the borders were corrected in axial and sagittal slices. The volume of the segmentation was calculated automatically by the software and expressed in cubic millimetres.

Supplementary Tables

Supplementary Table S1. Results of the group-wise comparison of for each amygdala nuclei and amygdala total volumes (ANCOVA)

**Amygdala volumes**	**Group mean (SD)**		**Model**		**Pedictors**		**Covariates**		
	**VPT**	**FT**	**R2**	**Corrected p-value (FDR)**	**Group**	**Group x Age**	**ICV**	**Sex**	**SES**
**Left-lateralised amygdala volumes**									
Lateral nucleus	670.36 (67.04)	676.74 (84.4)	0.41	q<.001	F(1,107)=0.702, p=0.404, η2=0.019	F(1,107)=0.003, p=0.96, η2=0	F(1,107)=43.216, p<.001, r=0.536	F(1,107)=3.822, p=0.053, r=0.186	F(1,107)=0.135, p=0.714, r=0.036
Basal nucleus	452.42 (44.67)	460.46 (58.87)	0.40	q<.001	F(1,107)=1.12, p=0.292, η2=0.007	F(1,107)=0.456, p=0.501, η2=0.004	F(1,107)=38.815, p<.001, r=0.516	F(1,107)=5.24, p=0.024, r=0.216	F(1,107)=0.011, p=0.918, r=0.01
Accessory basal nucleus	253.47 (29.3)	263.71 (33.85)	0.40	q<.001	F(1,107)=0.858, p=0.356, η2=0	F(1,107)=1.501, p=0.223, η2=0.014	F(1,107)=32.037, p<.001, r=0.48	F(1,107)=6.552, p=0.012, r=0.24	F(1,107)=0.77, p=0.382, r=0.085
Anterior amygdaloid area	60.72 (6.91)	59.67 (7.6)	0.21	q<.001	F(1,107)=0.59, p=0.444, η2=0.042	F(1,107)=0.269, p=0.605, η2=0.003	F(1,107)=18.892, p<.001, r=0.387	F(1,107)=0.266, p=0.607, r=0.05	F(1,107)=0.833, p=0.363, r=0.088
Central nucleus	42.43 (6.6)	45.25 (9.83)	0.29	q<.001	F(1,107)=2.16, p=0.145, η2=0.003	F(1,107)=4.467, p=0.037, η2=0.04	F(1,107)=12.571, p=0.001, r=0.324	F(1,107)=6.859, p=0.01, r=0.245	F(1,107)=1.478, p=0.227, r=0.117
Medial nucleus	20.15 (5.07)	21.09 (5.1)	0.10	q=.05	F(1,107)=0.153, p=0.696, η2=0	F(1,107)=0.342, p=0.56, η2=0.003	F(1,107)=6.497, p=0.012, r=0.239	F(1,107)=0.14, p=0.709, r=0.036	F(1,107)=0.717, p=0.399, r=0.082
Cortical medial nucleus	23.62 (3.82)	25.01 (3.42)	0.21	q<.001	F(1,107)=0.566, p=0.453, η2=0.006	F(1,107)=2.023, p=0.158, η2=0.019	F(1,107)=8.947, p=0.003, r=0.278	F(1,107)=2.737, p=0.101, r=0.158	F(1,107)=1.607, p=0.208, r=0.122
Cortico-amygdaloid transition area	177.48 (20.49)	183.39 (23.94)	0.42	q<.001	F(1,107)=1.105, p=0.296, η2=0	F(1,107)=1.93, p=0.168, η2=0.018	F(1,107)=49.656, p<.001, r=0.563	F(1,107)=1.205, p=0.275, r=0.106	F(1,107)=0.854, p=0.358, r=0.089
Paralaminar nuclei	52.14 (5.7)	52.64 (7.69)	0.37	q<.001	F(1,107)=0.321, p=0.572, η2=0.009	F(1,107)=0.002, p=0.968, η2=0	F(1,107)=32.94, p<.001, r=0.485	F(1,107)=5.603, p=0.02, r=0.223	F(1,107)=1.211, p=0.274, r=0.106
Whole amygdala volume	1752.8 (168.76)	1787.94 (218.23)	0.45	q<.001	F(1,107)=1.172, p=0.281, η2=0.007	F(1,107)=0.535, p=0.466, η2=0.005	F(1,107)=48.592, p<.001, r=0.559	F(1,107)=5.292, p=0.023, r=0.217	F(1,107)=0.121, p=0.729, r=0.034
**Right-lateralised amygdala volumes**									
Lateral nucleus	684.83 (67.11)	690.87 (83.87)	0.46	q<.001	F(1,107)=2.072, p=0.153, η2=0.031	F(1,107)=0.259, p=0.612, η2=0.002	F(1,107)=58.517, p<.001, r=0.595	F(1,107)=1.899, p=0.171, r=0.132	F(1,107)=1.989, p=0.161, r=0.135
Basal nucleus	455.22 (46.51)	466.09 (60.72)	0.45	q<.001	F(1,107)=0.979, p=0.325, η2=0.014	F(1,107)=0.144, p=0.705, η2=0.001	F(1,107)=55.858, p<.001, r=0.586	F(1,107)=1.256, p=0.265, r=0.108	F(1,107)=3.177, p=0.078, r=0.17
Accessory basal nucleus	261.72 (28.78)	273.24 (38.56)	0.47	q<.001	F(1,107)=0.162, p=0.689, η2=0	F(1,107)=0.142, p=0.707, η2=0.001	F(1,107)=53.695, p<.001, r=0.578	F(1,107)=3.714, p=0.057, r=0.183	F(1,107)=3.212, p=0.076, r=0.171
Anterior amygdaloid area	63.56 (8.23)	62.83 (8.14)	0.18	q<.001	F(1,107)=0.666, p=0.416, η2=0.03	F(1,107)=0.056, p=0.813, η2=0.001	F(1,107)=16.958, p<.001, r=0.37	F(1,107)=0.048, p=0.828, r=0.021	F(1,107)=1.834, p=0.179, r=0.13
Central nucleus	44.06 (6.95)	46.48 (6.44)	0.33	q<.001	F(1,107)=0.011, p=0.916, η2=0.006	F(1,107)=0.417, p=0.52, η2=0.004	F(1,107)=33.962, p<.001, r=0.491	F(1,107)=0.377, p=0.54, r=0.059	F(1,107)=0.273, p=0.602, r=0.05
Medial nucleus	22.2 (5.06)	23.16 (5.18)	0.11	q=.03	F(1,107)=1.113, p=0.294, η2=0.001	F(1,107)=2.036, p=0.157, η2=0.019	F(1,107)=7.367, p=0.008, r=0.254	F(1,107)=0.005, p=0.944, r=0.007	F(1,107)=0.026, p=0.872, r=0.016

Cortical medial nucleus	25.7 (3.57)	26.99 (4.19)	0.29	q<.001	F(1,107)=0.409, p=0.524, η2=0.002	F(1,107)=1.173, p=0.281, η2=0.011	F(1,107)=22.667, p<.001, r=0.418	F(1,107)=2.013, p=0.159, r=0.136	F(1,107)=0.241, p=0.625, r=0.047
Cortico-amygdaloid transition area	182.95 (21.86)	186.23 (25.96)	0.41	q<.001	F(1,107)=1.441, p=0.233, η2=0.015	F(1,107)=0.365, p=0.547, η2=0.003	F(1,107)=45.21, p<.001, r=0.545	F(1,107)=2.445, p=0.121, r=0.149	F(1,107)=1.105, p=0.296, r=0.101
Paralaminar nuclei	51.73 (5.55)	52.24 (7.19)	0.39	q<.001	F(1,107)=1.279, p=0.261, η2=0.021	F(1,107)=0.117, p=0.733, η2=0.001	F(1,107)=46.425, p<.001, r=0.55	F(1,107)=1.069, p=0.303, r=0.099	F(1,107)=0.12, p=0.729, r=0.034
Whole amygdala volume	1791.97 (174.48)	1828.13 (226.01)	0.49	q<.001	F(1,107)=1.446, p=0.232, η2=0.017	F(1,107)=0.303, p=0.583, η2=0.003	F(1,107)=65.864, p<.001, r=0.617	F(1,107)=2.165, p=0.144, r=0.141	F(1,107)=2.617, p=0.109, r=0.155

Notes: *q-value, corrected p-values using FDR; Effect sizes were calculated using η^2, Partial eta squared (partial η^2) for predictor variables and using r, r contrast for covariates*

Supplementary Table S2. PLSC results for the association between socio-emotional measures and amygdala volumes in the VPT and full-term control groups, corresponding to Figure 1. The table shows original socio-emotional saliences (bootstrap estimate standard deviation) and bootstrap ratio z-scores; as well as amygdala volume saliences (bootstrap estimate standard deviation) and bootstrap ratio z-scores.

Salience type: Socio-emotional measures	VPT		Full-term controls	
	Salience (bootstrap estimate standard deviation)	Bootstrap ratio z-scores	Salience (bootstrap estimate standard deviation)	Bootstrap ratio z-scores
Theory of Mind	-0.167 (0.116)	-1.43	0.101 (0.087)	1.169
Affect Recognition	-0.105 (0.13)	-0.803	-0.359 (0.118)	-3.034
Internalised Score	-0.054 (0.106)	-0.513	-0.194 (0.1)	-1.933
Emotional Control	0.847 (0.067)	12.695	0.255 (0.114)	2.235

Salience type: Amygdala subnuclei volumes	In both the VPT and full-term control groups	
	Salience (bootstrap estimate standard deviation)	Bootstrap ratio z-scores
Left Lateral nucleus	0.186 (0.068)	2.727
Left Basal nucleus	0.161 (0.066)	2.441
Left Accessory Basal nucleus	0.226 (0.064)	3.553
Left Anterior amygdaloid area	0.344 (0.075)	4.608
Left Central nucleus	0.14 (0.085)	1.639
Left Medial nucleus	0.003 (0.071)	0.044
Left Cortical nucleus	0.154 (0.084)	1.826
Left Corticoamygdaloid transitio	0.284 (0.074)	3.869
Left Paralaminar nucleus	0.079 (0.073)	1.075
Left Whole amygdala	0.213 (0.042)	5.101
Right Lateral nucleus	0.322 (0.057)	5.627
Right Basal nucleus	0.248 (0.05)	4.937
Right Accessory Basal nucleus	0.234 (0.055)	4.224
Right Anterior amygdaloid area	0.349 (0.079)	4.436
Right Central nucleus	-0.066 (0.086)	-0.767
Right Medial nucleus	-0.014 (0.08)	-0.173
Right Cortical nucleus	0.147 (0.074)	1.987
Right Corticoamygdaloid transitio	0.336 (0.061)	5.481
Right Paralaminar nucleus	0.174 (0.077)	2.252
Right Whole amygdala	0.29 (0.034)	8.608

Supplementary Table S3. PLSC results for the association between socio-emotional measures, adjusted for gestational age, and amygdala volumes in the VPT and full-term control groups, corresponding to Supplementary Figure S1. The table shows original socio-emotional saliences (bootstrap estimate standard deviation) and bootstrap ratio z-scores; as well as amygdala volume saliences (bootstrap estimate standard deviation) and bootstrap ratio z-scores.

Salience type: Socio-emotional measures	VPT		Full-term controls	
	Salience (bootstrap estimate standard deviation)	Bootstrap ratio z-scores	Salience (bootstrap estimate standard deviation)	Bootstrap ratio z-scores
Theory of Mind	-0.156 (0.117)	-1.333	0.09 (0.087)	1.035
Affect Recognition	-0.13 (0.129)	-1.006	-0.381 (0.11)	-3.467
Internalised Score	-0.104 (0.101)	-1.025	-0.14 (0.106)	-1.321
Emotional Control	0.829 (0.067)	12.41	0.298 (0.116)	2.574

Salience type: Amygdala subnuclei volumes	In both the VPT and full-term control groups	
	Salience (bootstrap estimate standard deviation)	Bootstrap ratio z-scores
Left Lateral nucleus	0.186 (0.067)	2.794
Left Basal nucleus	0.171 (0.059)	2.895
Left Accessory Basal nucleus	0.238 (0.064)	3.74
Left Anterior amygdaloid area	0.337 (0.078)	4.305
Left Central nucleus	0.146 (0.084)	1.747
Left Medial nucleus	0.015 (0.076)	0.192
Left Cortical nucleus	0.168 (0.079)	2.14
Left Corticoamygdaloid transitio	0.288 (0.07)	4.107
Left Paralaminar nucleus	0.09 (0.067)	1.333
Left Whole amygdala	0.219 (0.038)	5.748
Right Lateral nucleus	0.309 (0.057)	5.383
Right Basal nucleus	0.244 (0.051)	4.81
Right Accessory Basal nucleus	0.246 (0.056)	4.395
Right Anterior amygdaloid area	0.324 (0.076)	4.263
Right Central nucleus	-0.038 (0.084)	-0.455
Right Medial nucleus	0.018 (0.081)	0.223
Right Cortical nucleus	0.171 (0.071)	2.403
Right Corticoamygdaloid transitio	0.335 (0.063)	5.283

Right Paralaminar nucleus	0.174 (0.077)	2.259
Right Whole amygdala	0.287 (0.034)	8.534

Supplementary Table S4. PLSC results for the association between socio-emotional measures at school-age and amygdala volumes at TEA in the VPT subgroup for the non-significant LC1, p=0.475. The table shows original socio-emotional saliences (bootstrap estimate standard deviation) and bootstrap ratio z-scores; as well as amygdala volume saliences (bootstrap estimate standard deviation) and bootstrap ratio z-scores.

Salience type: Socio-emotional measures	VPT subgroup	
	Salience (bootstrap estimate standard deviation)	Bootstrap ratio z- scores
Theory of Mind	-0.421 (0.436)	-0.405
Affect Recognition	-0.754 (0.469)	-0.651
Internalised Score	-0.352 (0.363)	-0.332
Emotional Control	0.542 (0.393)	0.549
Salience type: Amygdala volumes	Salience (bootstrap estimate standard deviation)	Bootstrap ratio z- scores
Left amygdala	1.054 (0.459)	0.969
Right amygdala	0.298 (0.671)	0.244

Supplementary Table S5. PLSC results for the association between socio-emotional measures at school-age and longitudinal amygdala volume trajectories between TEA and school-age in the VPT subgroup for the non-significant LC1, p=0.387. The table shows original socio-emotional saliences (bootstrap estimate standard deviation) and bootstrap ratio z-scores; as well as amygdala volume saliences (bootstrap estimate standard deviation) and bootstrap ratio z-scores.

Salience type: Socio-emotional measures	VPT subgroup	
	Salience (bootstrap estimate standard deviation)	Bootstrap ratio z- scores
Theory of Mind	-0.505 (0.316)	-0.780

Affect Recognition	-0.319 (0.359)	-0.541
Internalised Score	0.072 (0.215)	0.159
Emotional Control	0.178 (0.276)	0.271
Salience type: Amygdala volumes	Salience (bootstrap estimate standard deviation)	Bootstrap ratio z- scores
Longitudinal left amygdala	0.635 (0.301)	0.822
Right amygdala	0.391 (0.312)	0.569

Supplementary Table S6. PLSC results for the association between socio-emotional measures, adjusted for gestational age, at school-age and amygdala volumes at TEA in the VPT subgroup for the non-significant LC1, p=0.210. The table shows original socio-emotional saliences (bootstrap estimate standard deviation) and bootstrap ratio z-scores; as well as amygdala volume saliences (bootstrap estimate standard deviation) and bootstrap ratio z-scores.

Salience type: Socio-emotional measures	VPT subgroup	
	Salience (bootstrap estimate standard deviation)	Bootstrap ratio z- scores
Theory of Mind	*-0.421 (0.436)*	*-0.405*
Affect Recognition	*-0.754 (0.469)*	*-0.651*
Internalised Score	*-0.352 (0.363)*	*-0.332*
Emotional Control	*0.542 (0.393)*	*0.549*
Salience type: Amygdala volumes	Salience (bootstrap estimate standard deviation)	Bootstrap ratio z- scores
Left amygdala	*1.054 (0.459)*	*0.969*
Right amygdala	*0.298 (0.671)*	*0.244*

Supplementary Table S7. PLSC results for the association between socio-emotional measures, adjusted for gestational age, at school-age and longitudinal amygdala volume trajectories between TEA and school-age in the VPT subgroup for the non-significant LC1, p=0.228. The table shows original socio-emotional saliences (bootstrap estimate standard deviation) and bootstrap ratio z-scores; as well as amygdala volume saliences (bootstrap estimate standard deviation) and bootstrap ratio z-scores.

Salience type: Socio-emotional measures	VPT subgroup	
	Salience (bootstrap estimate standard deviation)	Bootstrap ratio z- scores
Theory of Mind	-0.505 (0.316)	-0.780
Affect Recognition	-0.319 (0.359)	-0.541
Internalised Score	0.072 (0.215)	0.159
Emotional Control	0.178 (0.276)	0.271
Salience type: Amygdala volumes	Salience (bootstrap estimate standard deviation)	Bootstrap ratio z- scores
Longitudinal left amygdala	0.635 (0.301)	0.822
Right amygdala	0.391 (0.312)	0.569

Supplementary Figures

Left-lateralised

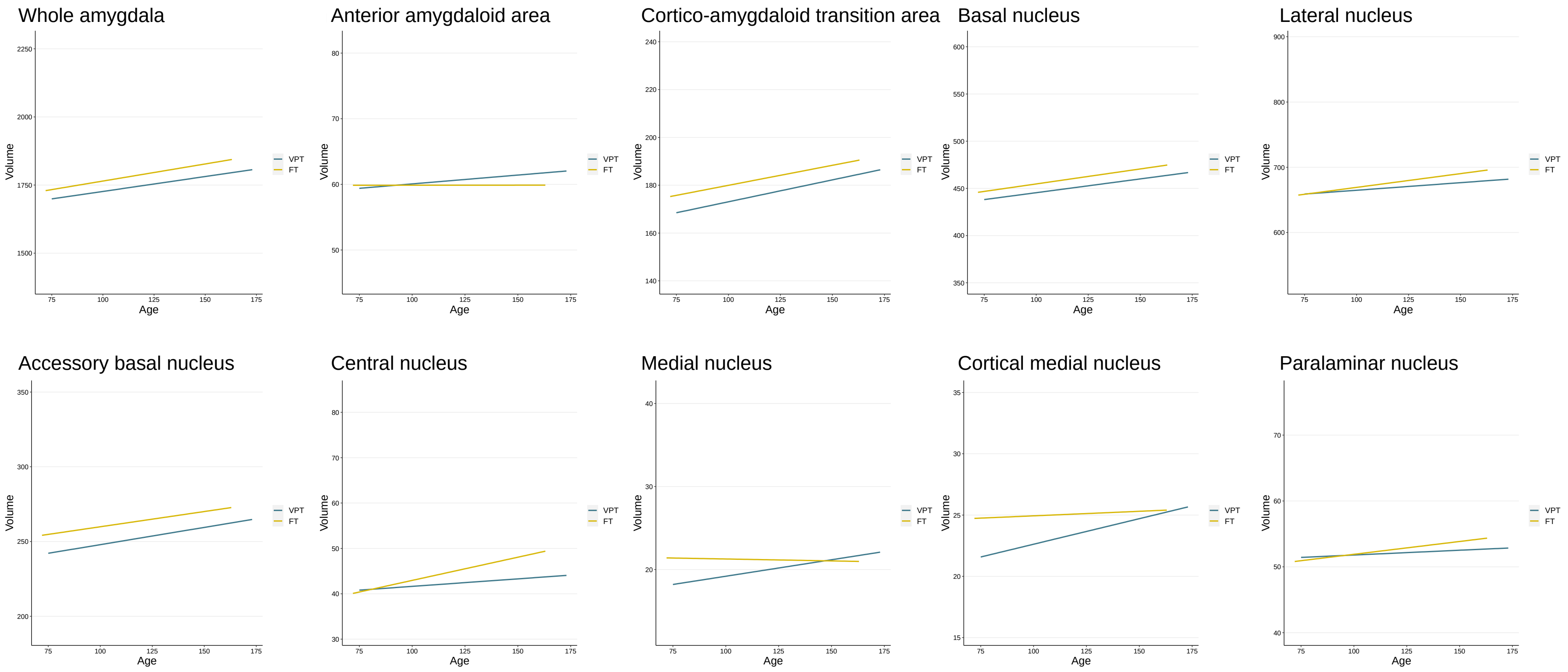

Supplementary Figure S1. Visualisation of left-lateralised amygdala volumes for each left amygdala nuclei and left amygdala total volume across groups and age (in months)

Right-lateralised

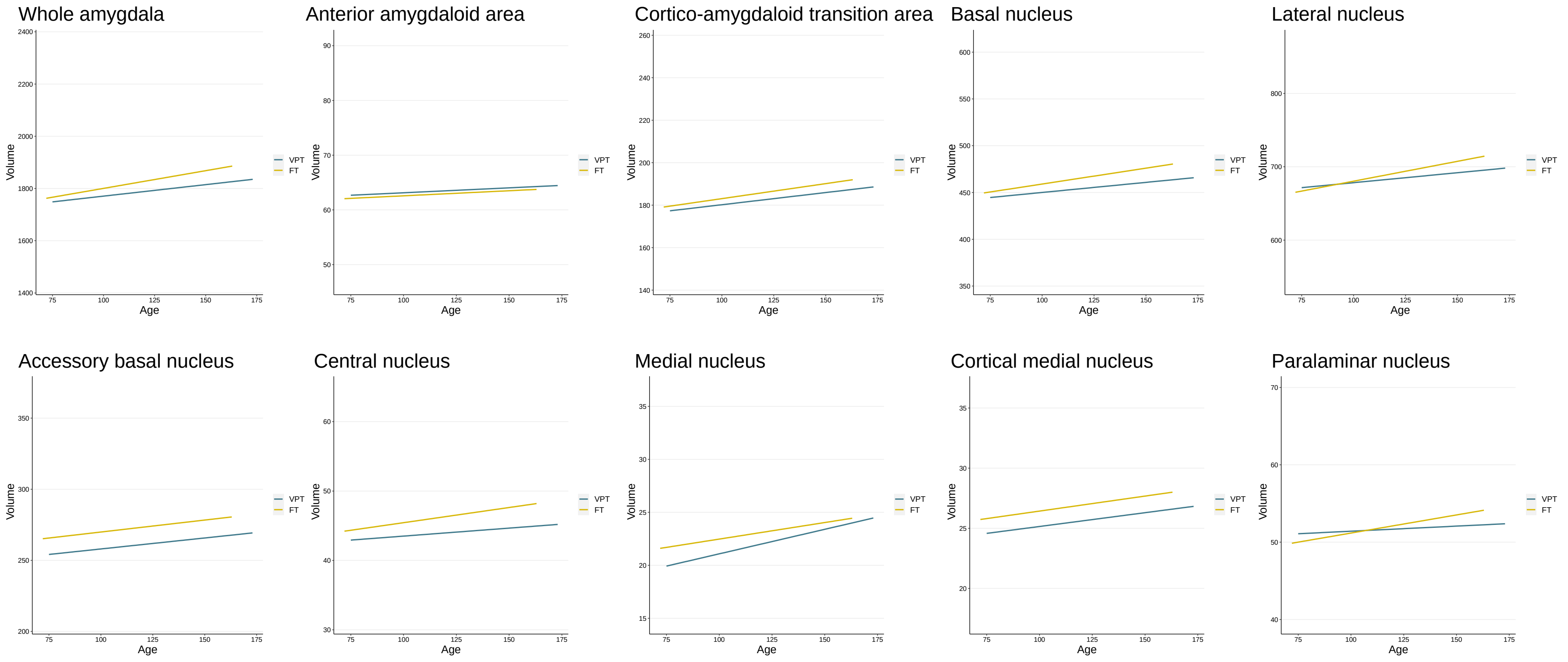

Supplementary Figure S2. Visualisation of right-lateralised amygdala volumes for each right amygdala nuclei and right amygdala total volume across groups and age.

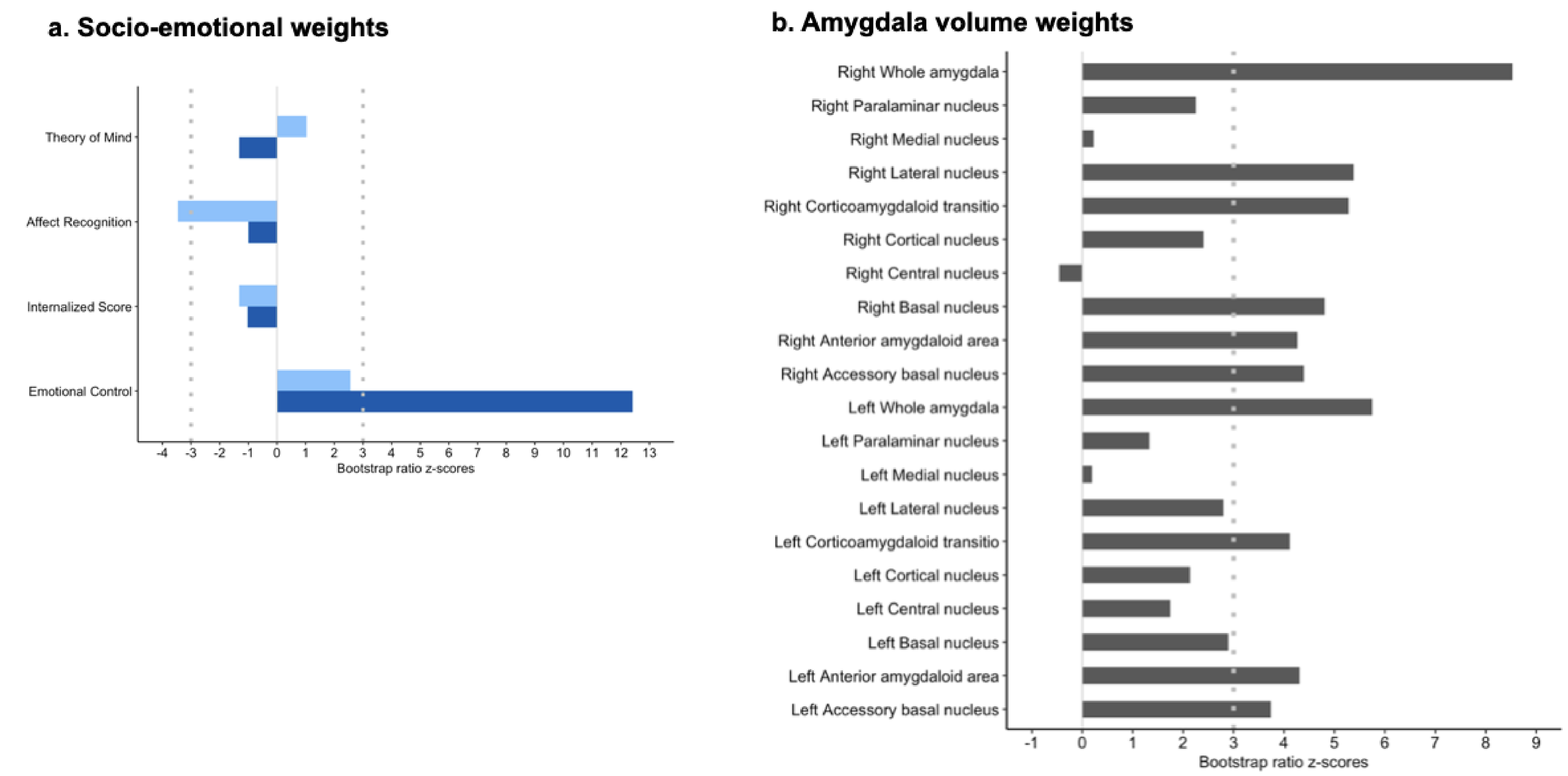

Supplementary Figure S3. Associations between amygdala volumetric measures and socio-emotional competencies at school-age in the very preterm (VPT) and full-term (FT) groups, were adjusted for age at testing, socio-economic status and gestational age, based on the PLSC analysis. Each latent component is composed of a set of socio-emotional weights (different for each group) and amygdala volume weights (common to all participants), which indicate how strongly each socio-emotional and amygdala volume variable contribute to the multivariate socio-emotional/amygdala volume association. a) The diverging graph shows socio-emotional bootstrap ratio z-scores for the VPT and the FT groups in dark blue and in light blue, respectively. Socio-emotional measures with an absolute

bootstrap ratio z-score ≥ 3 or ≤ -3 yield a robust contribution to the component. b) The diverging graph shows amygdala volume bootstrap ratio z-scores. Amygdala volumes with an absolute bootstrap ratio z- score ≥ 3 or ≤ -3 yield a robust contribution to the component.

A)

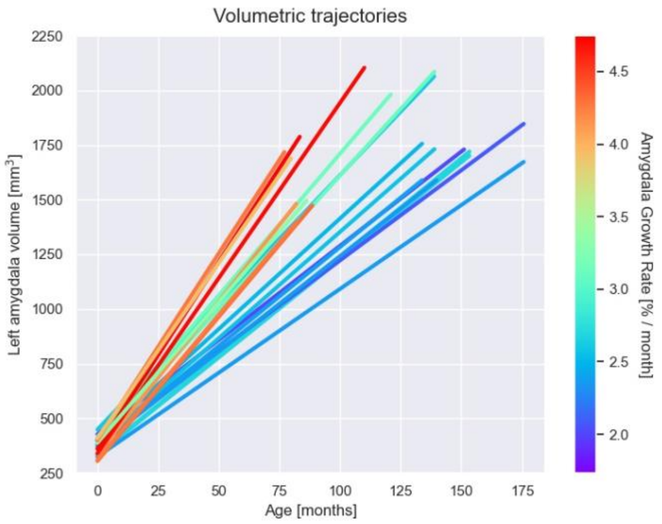

B)

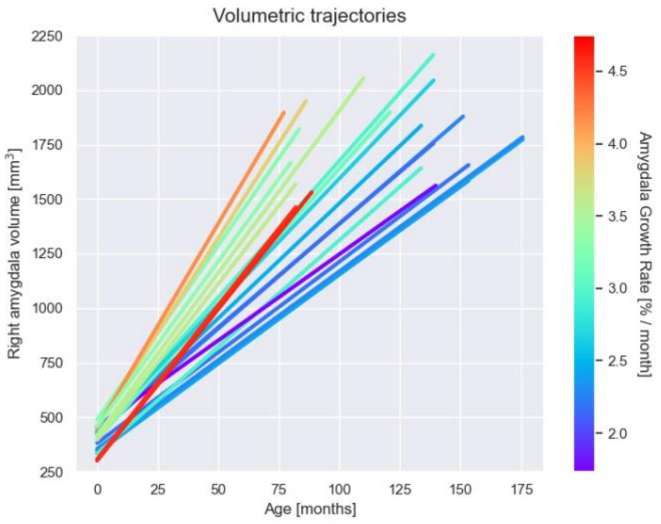

Supplementary Figure S4. A) Individual volume growth trajectories of the left amygdala from TEA to school-age. X-axis: age in months; Y-axis: total left amygdala volume in mm^3. B) Individual volume growth trajectories of the right amygdala from TEA to school-age. X-axis: age in months; Y-axis: total right amygdala volume in mm^3. Line colors indicate the individual's amygdala growth rate relative to the amygdala volume at TEA (faster and slower growth are represented by red and blue colors, respectively).

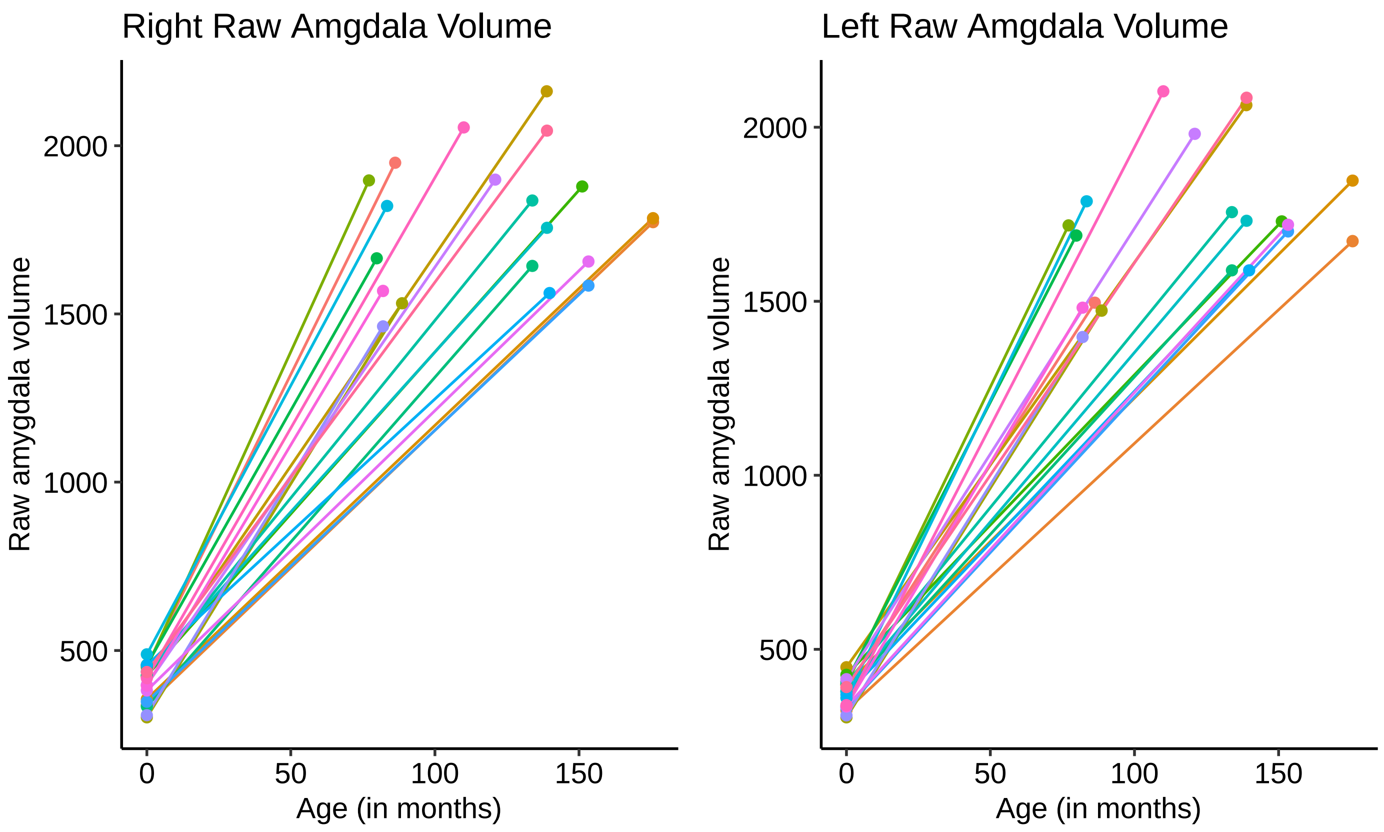

Supplementary Figure S5. Raw amygdala volume (left and right) in relation to age at MRI (in month), including MRI at TEA and MRI at school-age.

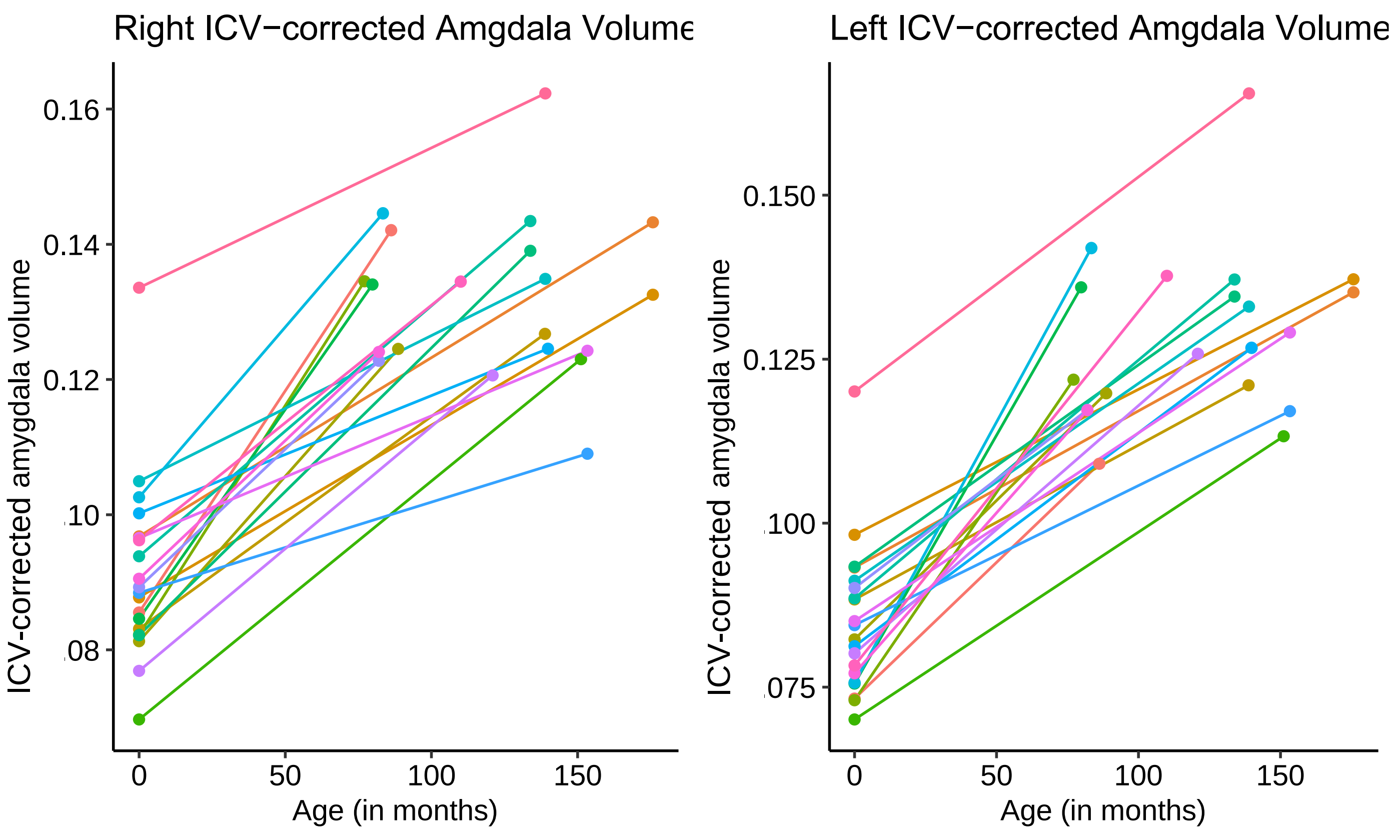

Supplementary Figure S6. ICV-corrected amygdala volume (left and right) in relation to age at MRI (in month), including MRI at TEA and MRI at school-age.

REFERENCES

1. Largo RH, Pfister D, Molinari L, Kundu S, Lipp A, Duc G. Significance of prenatal, perinatal and postnatal factors in the development of AGA preterm infants at five to seven years. Dev Med Child Neurol [Internet] 1989;31:440–56. Available from: https://www.ncbi.nlm.nih.gov/pubmed/2680687
2. Reynolds CR, Kamphaus RW, Rosenthal BL. Applications of the Kaufman Assessment Battery for Children (K-ABC) in neuropsychological assessment. In: Springer, editor. Handbook of clinical child neuropsychology (3rd ed.). NY: 2009.
3. Wechsler D. Wechsler Intelligence Scale for Children (5th ed.). Bloomington, MN: PsychCorp; 2014.
4. Korkman M, Kirk U, Kemp S. NEPSY-Second Edition (NEPSY-II). J Psychoeduc Assess [Internet] 2007 [cited 2021 Dec 27];28:175–82. Available from: http://jpa.sagepub.com
5. Goodman R. The Strengths and Difficulties Questionnaire: A Research Note. Journal of Child Psychology and Psychiatry [Internet] 1997 [cited 2022 Dec 27];38:581–6. Available from: https://onlinelibrary.wiley.com/doi/full/10.1111/j.1469-7610.1997.tb01545.x
6. Goodman R. Psychometric Properties of the Strengths and Difficulties Questionnaire. J Am Acad Child Adolesc Psychiatry 2001;40:1337–45.
7. Gioia GA, Isquith PK, Guy SC, Kenworthy L. Overview & What's New Behavior Rating Inventory of Executive Function ® , Second Edition (BRIEF ® 2) Test Materials • BRIEF2 Professional Manual with Fast Guide.
8. Tamnes CK, Roalf DR, Goddings AL, Lebel C. Diffusion MRI of white matter microstructure development in childhood and adolescence: Methods, challenges and progress. Dev Cogn Neurosci 2018;33:161–75.
9. de Bie HMA, Boersma M, Wattjes MP, et al. Preparing children with a mock scanner training protocol results in high quality structural and functional MRI scans. Eur J Pediatr [Internet] 2010 [cited 2022 Dec 27];169:1079–85. Available from: https://link.springer.com/article/10.1007/s00431-010-1181-z
10. Gui L, Loukas S, Lazeyras F, Hüppi PS, Meskaldji DE, Borradori Tolsa C. Longitudinal study of neonatal brain tissue volumes in preterm infants and their ability to predict neurodevelopmental outcome. Neuroimage 2019;185:728–41.
11. Mai JK, Paxinos G. The Human Nervous System, Third Edition. The Human Nervous System, Third Edition [Internet] 2012 [cited 2024 Jan 22];1–1415. Available from: http://www.sciencedirect.com:5070/book/9780123742360/the-human-nervous-system
12. Bayer SA, Altman J. The Human Brain During the Third Trimester. The Human Brain During the Third Trimester 2003
